# Supplementary material for: Where Top-Down Meets Bottom-Up: Cell-Type Specific Connectivity Map of the Whisker System
Source: Neuroinformatics. 2024 May 20;22(3):251–68. doi: 10.1007/s12021-024-09658-6 (PMC11329691; doi:10.1007/s12021-024-09658-6)
Supplement: Supplementary file 1 — (pdf 301396 KB) [file 12021_2024_9658_MOESM1_ESM.pdf]

SupplementaryMaterials

TG line	Classification	Source
A930038C07Rik-Tg1-Cre	Excitatory	Harris et al., 2014
Adycap1-2A-Cre	Uncategorized	Zeisel et al., 2018
Calb2-IRES-Cre	Inhibitory	Taniguchi et al., 2011
Calb1-T2A-dgCre	Inhibitory	Wang et al., 2023
Cart-IRES2-Cre	Uncategorized	Zeisel et al., 2018
Cart-Tg1-Cre	Uncategorized	Zeisel et al., 2018
Cck-IRES-Cre	Uncategorized	Taniguchi et al., 2011
Chat-IRES-Cre-neo	Uncategorized	Zeisel et al., 2018
Chrna2-Cre-OE25	Inhibitory	Leão et al., 2012
Cort-T2A-Cre	Inhibitory	Taniguchi et al., 2011
ctgf-T2A-dgCr	Excitatory	Tasic et al., 2016
Cux2-CreERT2	Excitatory	Zeisel et al., 2018
Drd3-Cre-KI196	Inhibitory	Volta et al., 2011
Efr3a-Cre-NO108	Uncategorized	Zeisel et al., 2018
Erbb4-T2A-CreERT2	Inhibitory	Madisen et al., 2010
Gabrr3-Cre-KC112	Inhibitory	Parolari 2020
Gad2-IRES-Cre	Inhibitory	Taniguchi et al., 2011
Gal-Cre-KI87	Excitatory	Zeisel et al., 2018
Grik4-Cre	Uncategorized	Zeisel et al., 2018
Htr2a-Cre-KM20	Excitatory	Rang et al., 2015
Lypd6-Cre-KL156	Uncategorized	Zeisel et al., 2018
Nos1-CreErt2	Inhibitory	Taniguchi et al., 2011
Npr3-IRES2-Cre	Excitatory	Daigle et al., 2018
Ntrk1-IRES-Cre	Uncategorized	Zeisel et al., 2018
Ntsr1-Cre-GN220	Excitatory	Madisen et al., 2010
Oxtr-Cre-ON66	Uncategorized	Zeisel et al., 2018
Oxtr-T2A-Cre	Uncategorized	
Pdzk1ip1-Cre-KD31	Uncategorized	
Plxnd-Cre-OG	Excitatory	Zeisel et al., 2018
Ppp1r17-Cre-NL1	Inhibitory	Zeisel et al., 2018
Prkcd-GluCla-CFP-IRES	Uncategorized	Zeisel et al., 2018
Pvalb-IRES-Cre	Inhibitory	Madisen et al., 2010
Rasgrf2-T2A-dCre	Uncategorized	Stacey et al., 2012
Rbp4-Cre-Kl100	Excitattory	Zeisel et al., 2018
Rorb-IRES-Cre	Uncategorized	Zeisel et al., 2018
Scnn1a-Tg3-Cre	Excitatory	Madisen et al., 2010
Sepw1-Cre-NP39	Uncategorized	Zeisel et al., 2018
Sim1-Cre-KJ18	Excitatory	Zeisel et al., 2018
Slc6a3-Cre	Uncategorized	Zeisel et al., 2018
Slc6a4-Cre-ET33	Uncategorized	Zeisel et al., 2018
Slc6a4-CreERT2-EZ13	Uncategorized	Zeisel et al., 2018
Slc6a5-Cre-KF10	Inhibitory	Morrow et al., 1998
Slc17a6-IRES-Cre	Excitatory	Harris et al., 2014
Slc17a8-IRES2-Cre	Excitatory	Tasic et al., 2018
Slc18a2-Cre-OZ14	Excitatory	Tritsch et al., 2014

Slc32a1-IRES-Cre	Inhibitory	Harris et al., 2014
Sst-IRES-Cre	Inhibitory	Taniguchi et al., 2011
Syt6-Cre-KI148	Uncategorized	Safran et al., 2010
Syt17-Cre-NO14	Excitatory	Ruhl et al., 2019
Tlx3-Cre-PL56	Excitatory	Cheng et al. 2004
Th-Cre-FI172	Uncategorized	Uhlèn et al., 2015

Supplemental Table 1: Type of neuron marked by each transgenic line

structure	wild type experiments	transgenic line experiments	transgenic lines	expressed in number of structures	transgenic line showing connections
MOp	8	21	15	13	12
ZI	4	15	13	11	11
PO	3	6	3	3	3
bfd	6	40	29	21	16
PSV	2	1	2	1	1
VTA	2	12	12	9	8
SI	7	10	8	7	7
SPVO	1	0	0	0	0
LC	0	0	0	0	0
SC	7	14	9	7	7
TM	0	1	1	0	0
VPM	3	6	6	5	5
VII	2	5	4	3	3
PPN	0	3	3	2	2
SPVC	3	6	6	5	5
SPVI	2	2	1	1	1
RT	2	11	7	7	7
DR	0	9	5	4	4

Supplemental Table 2: For each structure, this table presents the number of experiments ran (transgenic and wild), the number of transgenic lines tested, the number of transgenic lines showing marked neurons in the considered structures and the number of transgenic lines showing a connection.

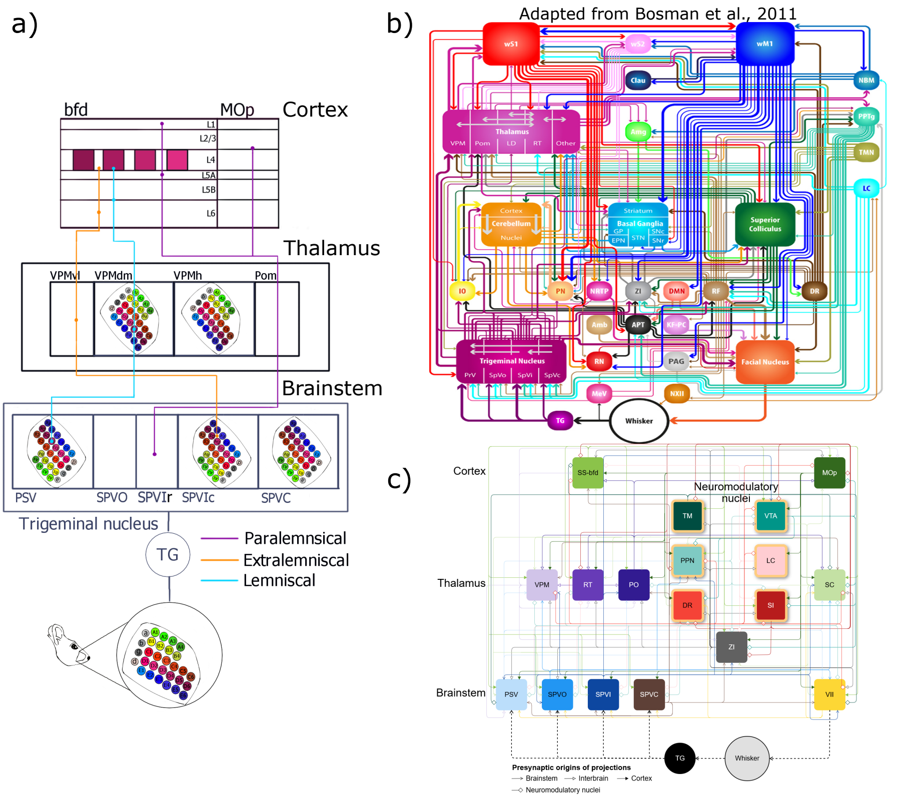

Supplemental Fig. 1 a) The whisker pad is organized in a grid-like structure. Each whisker is represented in SPVI, PSV, VPM, and bfd by an agglomerate of neurons organized in a barrel-like shape, respecting the coordinates of the whisker on the whisker pad with rotations. The information from the whisker enters the system through the trigeminal ganglion, projecting to the trigeminal nuclei. Here the information flow is divided into three major pathways: 1) the lemniscal pathway (blue), originating from the barrelettes of PSV, ascending to the barreloids of VPMdm and ending in the barrels of the layer 4 of the somatosensory pathway; 2) the extralemniscal pathway (orange), taking its origin in inter-barrelette neurons of SPVI, connecting to VPMvl and targeting the primary somatosensory cortex; and 3) the paralemniscal pathway (purple), taking its source in SPVI, targeting PO, which then targets MOp and bfd. b) The connectivity map presented by Bosman et al. (2011).The 18 nuclei that we focus in our study present 72 connections in the previous map. c) New connectome composed of 157 connections.

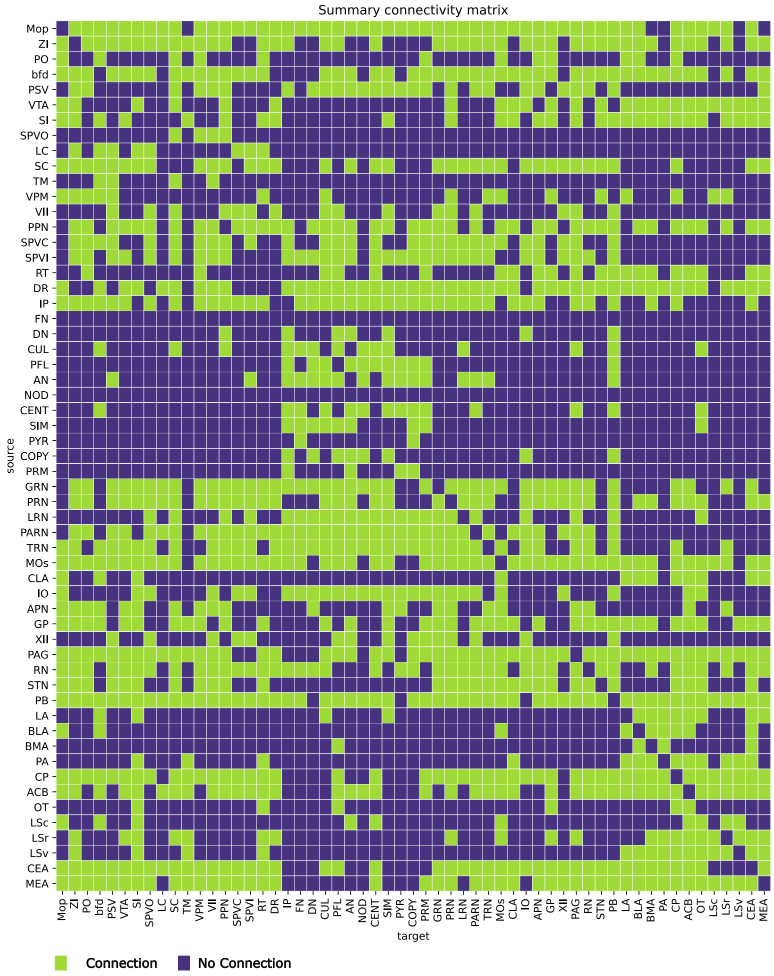
Summary connectivity matrix
source
target
Mop
ZI
PO
bfd
PSV
VTA
SI
SPVO
LC
SC
TM
VPM
VII
PPN
SPVC
SPVI
RT
DR
IP
FN
DN
CUL
PFL
AN
NOD
CENT
SIM
PYR
COPY
PRM
GRN
PRN
LRN
PARN
TRN
MOs
CLA
IO
APN
GP
XII
PAG
RN
STN
PB
LA
BLA
BMA
PA
CP
ACB
OT
LSc
LSr
LSv
CEA
MEA
Connection
No Connection

Supplemental Fig. 2 Summary of connection between 57 nuclei. **MOp**: Primary Motor Cortex, **Zi**: Zona Incerta, **PO**: Posterior complex of the thalamus, **bfd**: Barrel field of the primary somatosensory cortex, **PSV**: nucleus principalis of the trigeminal nucleus, **VTA**: Ventral tegmental area, **SI**: Substantia Innominata, **SPVO**: Spinal nucleus of the trigeminal nucleus oral part, **LC**: Locus coeruleus, **SC**: Superior Colliculus, **TM** : Tuberommaimillary Nucleus, **VPM**: Ventraposteromedial nucleus of the thalamus, **VII**: Facial motor nucleus, **PPN**: Pedunculopontine nucleus, **SPVC**: Spinal nucleus of the trigeminal nucleus caudal part, **SPVI**: Spinal nucleus of the trigeminal nucleus interoplaris part, **RT**: Reticular nucleus of the thalamus, **DR**: Dorsal Raphe nucleus, **IP**: Interposed nucleus, **FN**: Fastigial nucleus, **DN**: Dentate nucleus, **CUL**: Culmen, **PFL**: Paraflocculus, **AN**: Ansiform lobule, **NOD**: Nodulus(X), **CENT**: Central lobule, **SIM**: Simple lobule, **PYR**: Pyramus(VIII), **COPY**: Copula pyramidis, **PRM**: Paramedian lobule, **GRN**: Gigantocellular reticular nucleus, **PRN**: Pontine reticular nucleus, **LRN**: Lateral reticular nucleus, **PARN**: Parvicellular reticular nucleus, **TRN**: Tegmental reticular nucleus, **MOs**: Secondary motor area, **CLA** : Claustrum, **IO**: Inferior olivary complex, **APN**: Anterior pretectal nucleus, **GP**: Globus Pallidus, **XII**: Hypoglossal nucleus, **PAG**: Periaqueductal gray, **RN**: Red nucleus, **STN**: Subthalamic nucleus, **PB**: Parabrachial nucleus, **LA**: Lateral amygdalar nucleus, **BLA**: Basolateral amygdalar nucleus, **BMA**: Basomedial amygdalar nucleus, **PA**: Posterior amygdalar nucleus, **CP**: Caudoputamen, **ACB**: nucleus accumbens, **OT**: Olfactory tubercle, **LSc**: Lateral Septal nucleus caudal part, **LSr**: Lateral Septal nucleus rostral part, **LSv**: Lateral Septal nucleus ventral part, **CEA**: Central amygdalar nucleus, **MEA**: Medial amygdalar nucleus

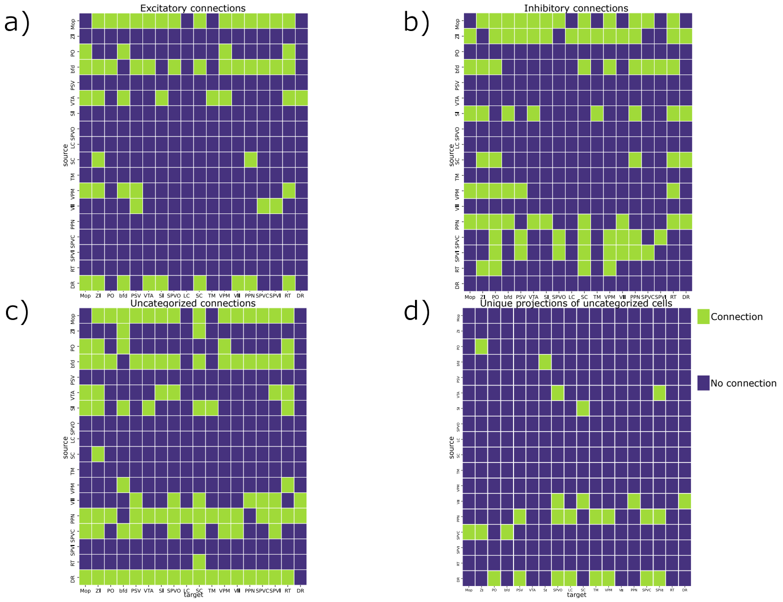

Supplemental Fig. 3 **a)** The excitatory connectivity matrix contains 58 connections. **b)** The inhibitory connectivity matrix contains 84 connections. **c)** 101 connections with uncategorized presynaptic cells were unveiled. **d)**27 connections were shown only by uncategorized transgenic-line

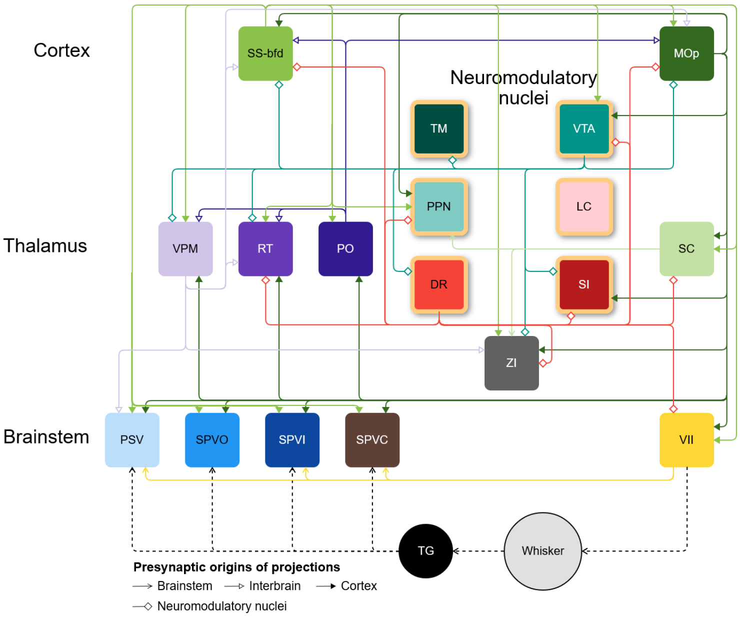

Supplemental Fig. 4 The excitatory connectivity map contains 58 connections. The dashed line represents the flow of information outside of the central nervous system. The neuromodulatory structures are represented with yellow borders.

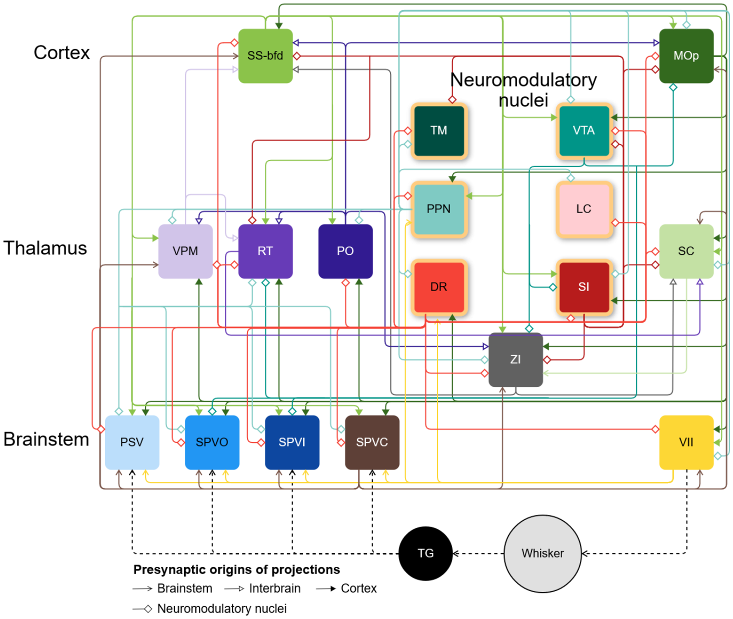

Supplemental Fig. 5 101 connections were revealed by uncategorized transgenic lines. The dashed line represents the flow of information outside of the central nervous system. The neuromodulatory structures are represented with yellow borders.

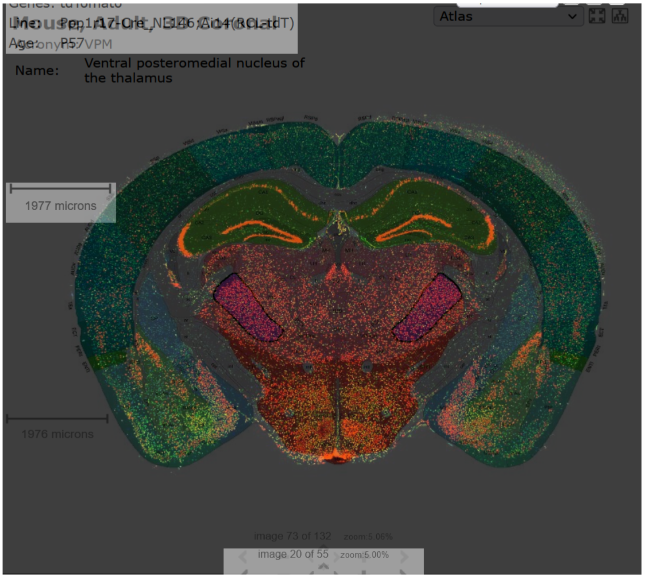

Supplemental Fig. 6 Expression in VPM of the transgenic lines PPP1r17 (inhibitory)

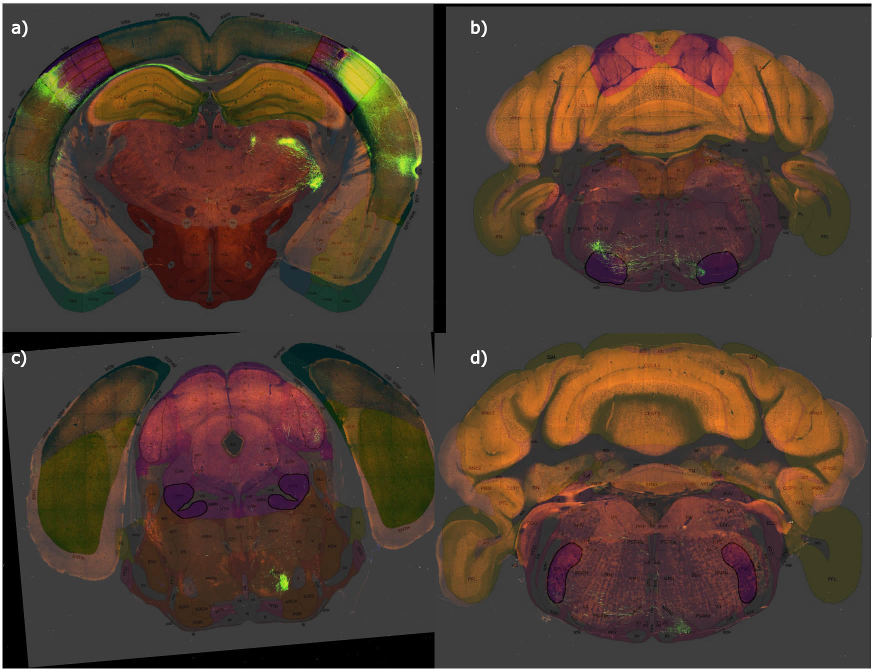

Supplemental Fig. 7 **a)** Injection structure bfd to **b)** VII, **c)** PPN, and **d)** SPVo. Experiment number 112951804, wild-type. The shortcomings of experimental procedures are addressed in the discussion.

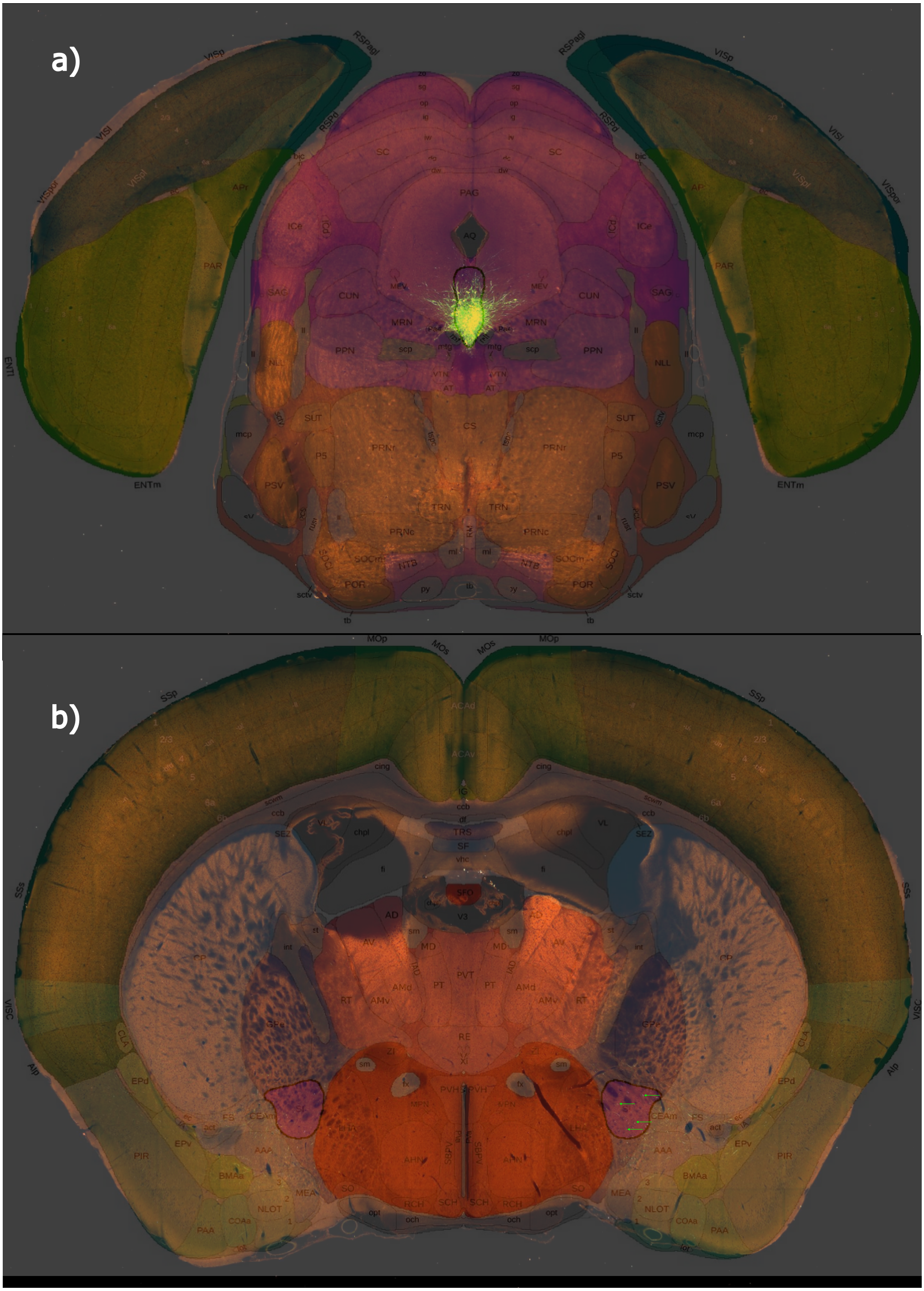

Supplemental Fig. 8 **a)** Injection structure DR to **b)** SI. Experiment number 582609848, line Esr2-IRES2-Cre. The shortcomings of experimental procedures are addressed in the discussion.

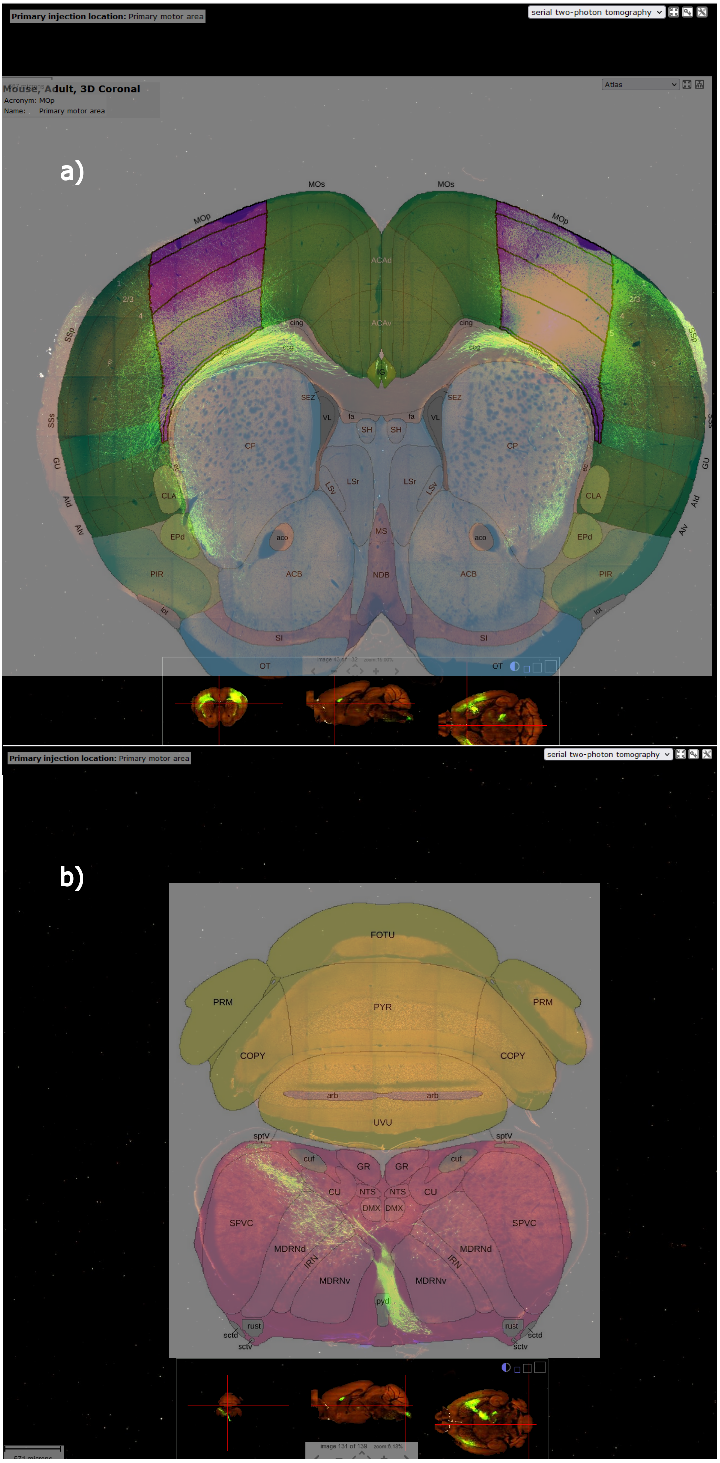

Supplemental Fig. 9 **a)** Injection structure MOp to **b)** SPVc. Experiment number 100141780, wild-type. The shortcomings of experimental procedures are addressed in the discussion.

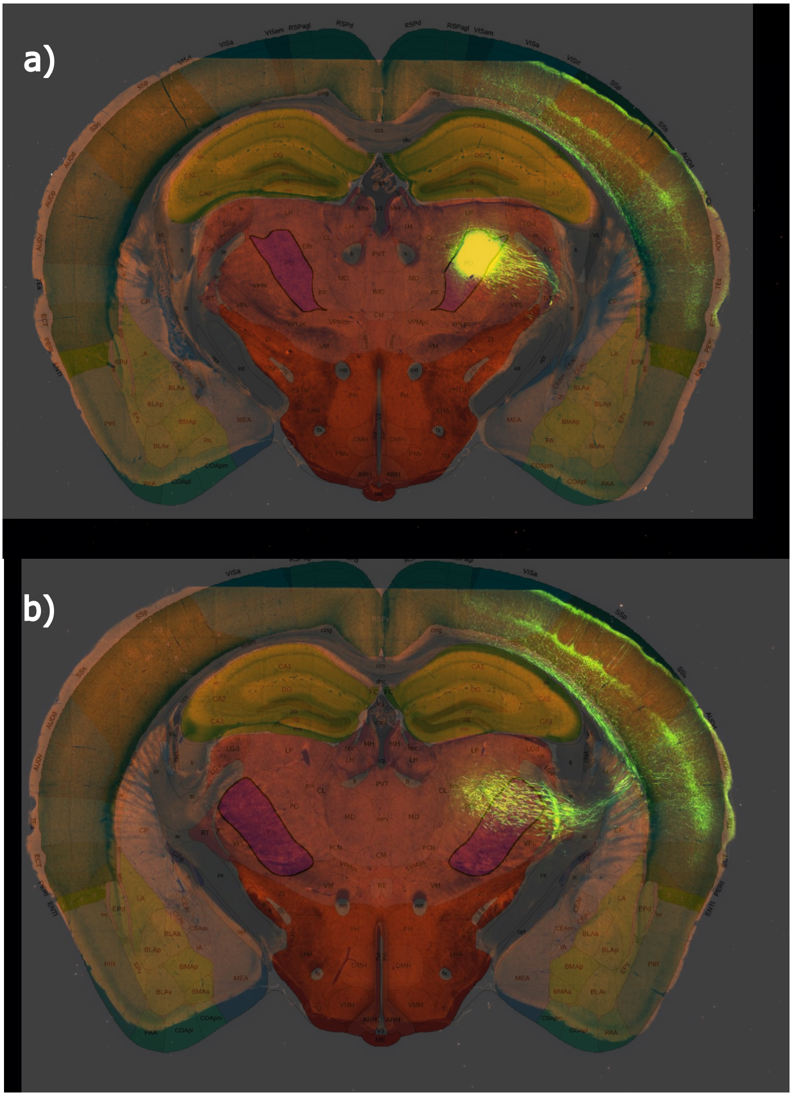

Supplemental Fig. 10 a) Injection structure PO to **b)** VPM. Experiment number 183011353, line Grik4-Cre. The shortcomings of experimental procedures are addressed in the discussion.

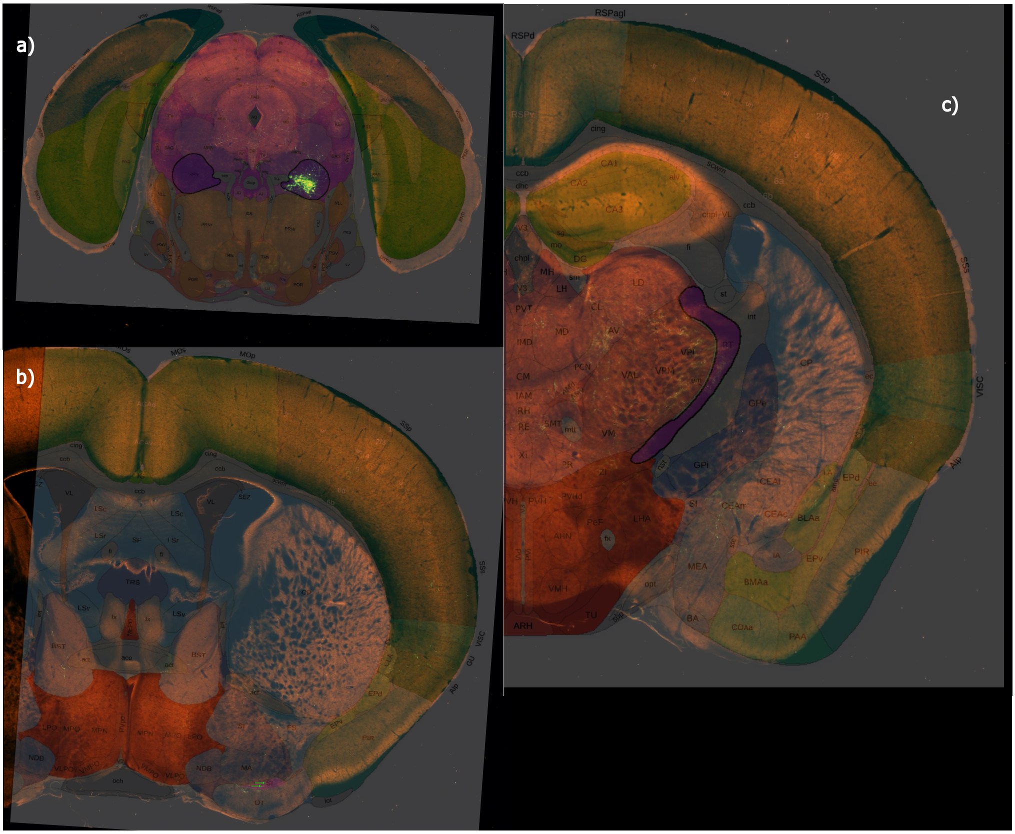

Supplemental Fig. 11 **a)** Injections structure PPN to **b)** SI and **c)** RT. Experiment number 264566672, line Chat-IRES-Cre-neo. The shortcomings of experimental procedures are addressed in the discussion.

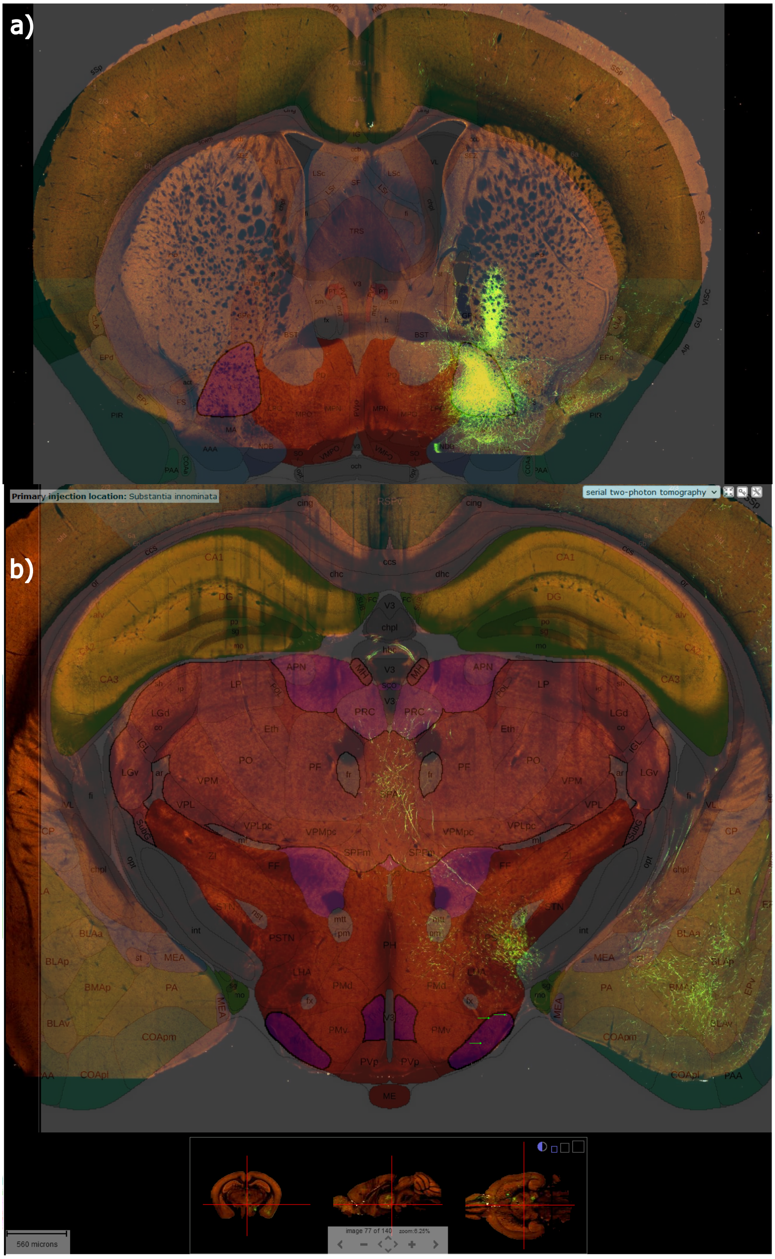

Supplemental Fig. 12 a) Injection structure SI to **b)** TM. Experiment number 305026861, line Drd3-Cre-KI196. The shortcomings of experimental procedures are addressed in the discussion.

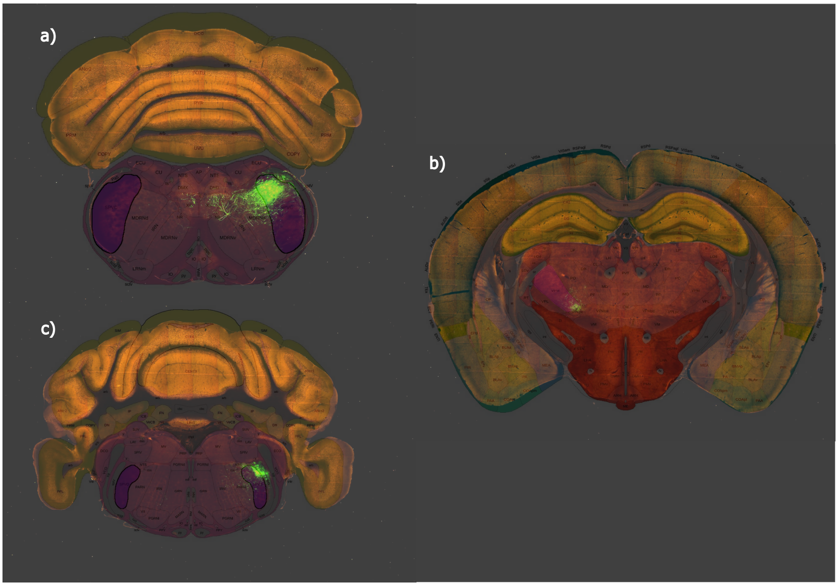

Supplemental Fig. 13 **a)** Injection structure SPVc to **b)** VPM and **c)** SPVo. Experiment number 114402050, wild-type. The shortcomings of experimental procedures are addressed in the discussion.

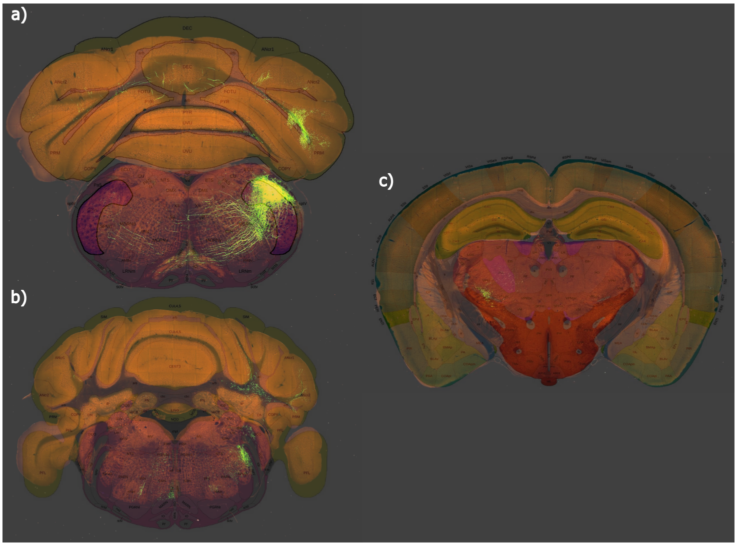

Supplemental Fig. 14 **a)** Injection structure SPVi to **b)** SPVo and **c)** PO. Experiment number 272738620, wild-type. The shortcomings of experimental procedures are addressed in the discussion.

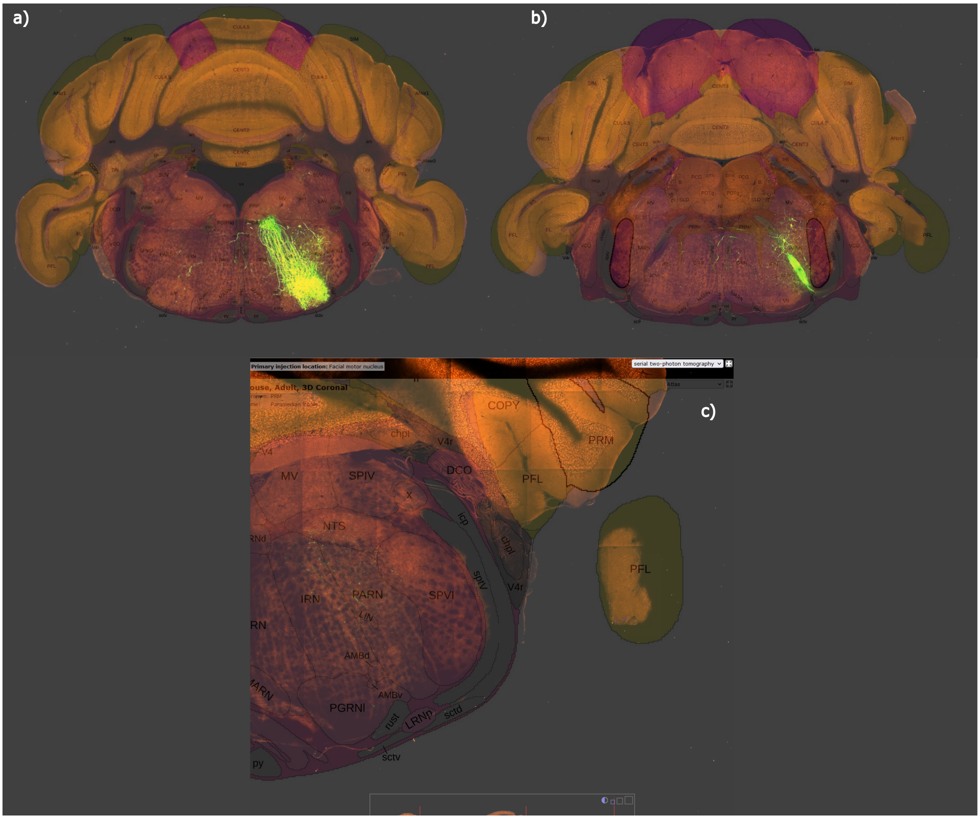

Supplemental Fig. 15 **a)** Injection structure VII to SPVo **b)** PSV, and **c)** to SPVi. Experiment number 177905562, line Scnn1a-Tg2-Cre. The shortcomings of experimental procedures are addressed in the discussion.

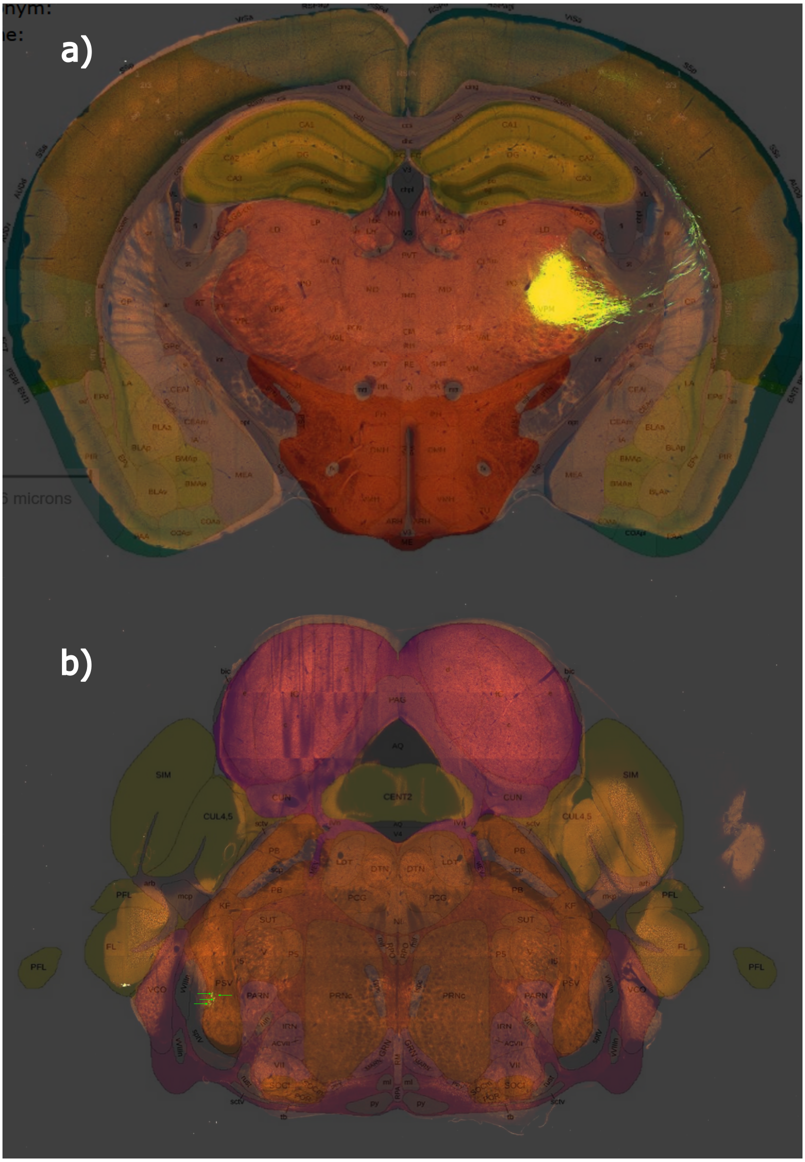

Supplemental Fig. 16 a) Injection structure VPM to **b)** PSV, experiment number 312240825, line Slc17a6-IRES-Cre. The shortcomings of experimental procedures are addressed in the discussion.

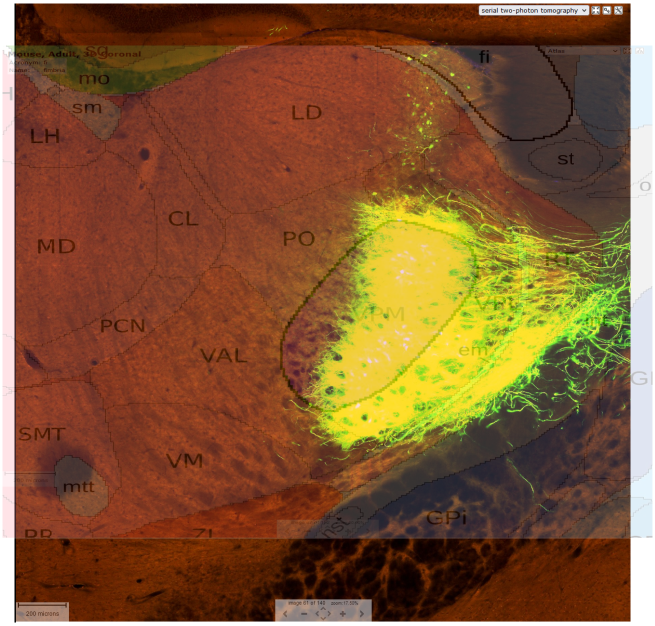

Supplemental Fig. 17 Inhibition target and source VPM to PO, experiment number 268206050, line Ppp1r17-Cre-NL146. The shortcomings of experimental procedures are addressed in the discussion.

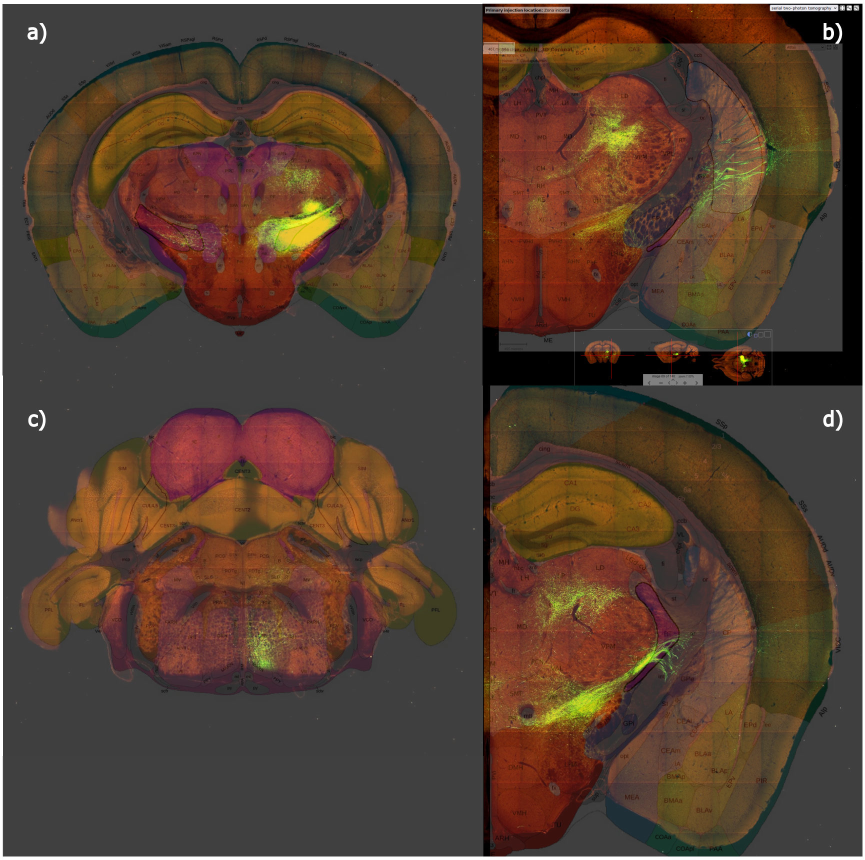

Supplemental Fig. 18 **a)** Injection structure ZI to **b)** SI, **c)** PSV, **d)** RT. Experiment number 113095845, wild-type. The shortcomings of experimental procedures are addressed in the discussion.

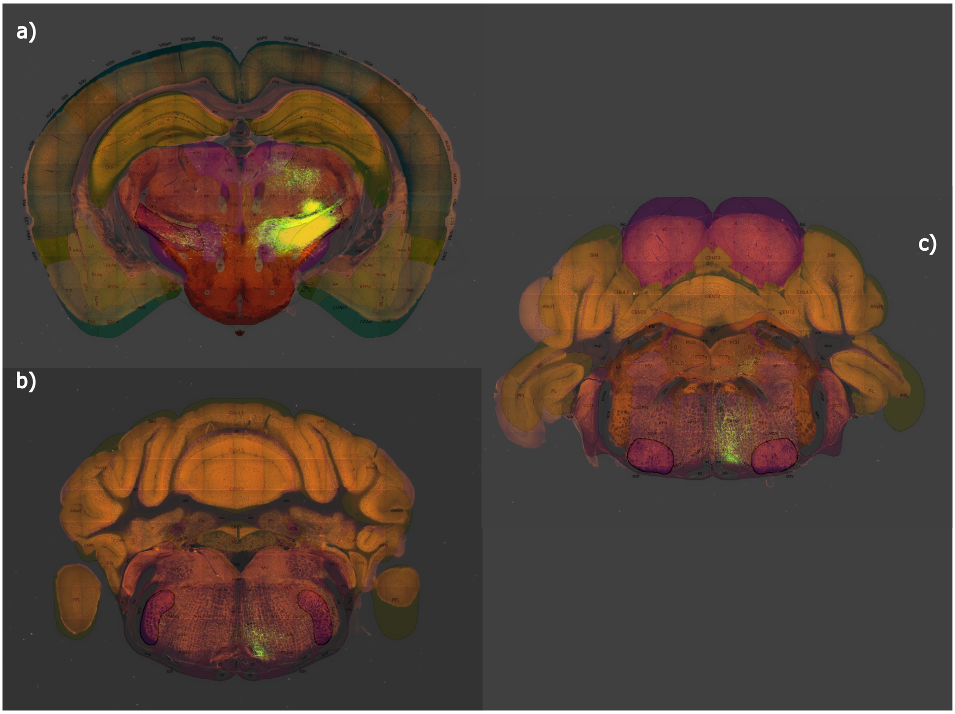

Supplemental Fig. 19 **a)** Injection structure ZI to **b)** SPVo, and **c)** VII. Experiment number 113095845, wild-type. The shortcomings of experimental procedures are addressed in the discussion.

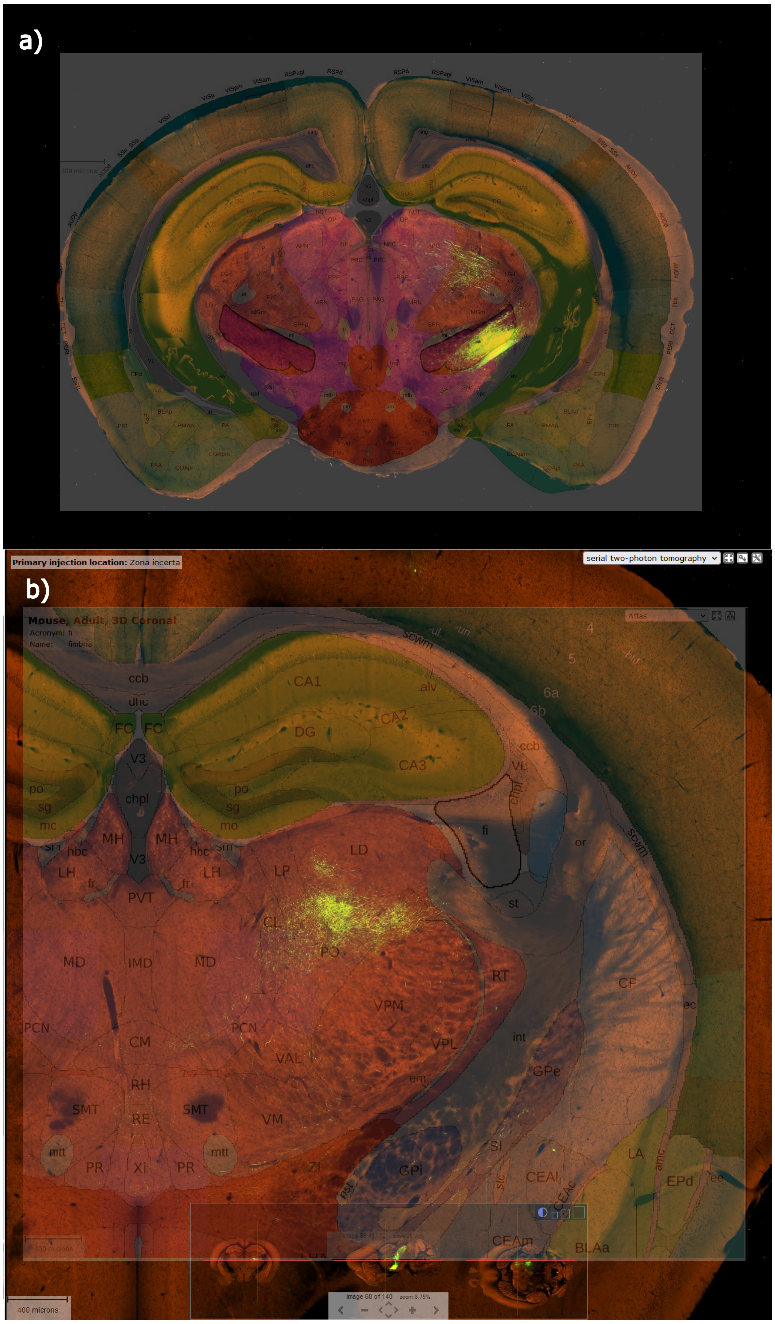

Supplemental Fig. 20 a) Injection structure ZI to **b)** VPM. Experiment number 301539438, line Pvalb-IRES-Cre. The shortcomings of experimental procedures are addressed in the discussion.

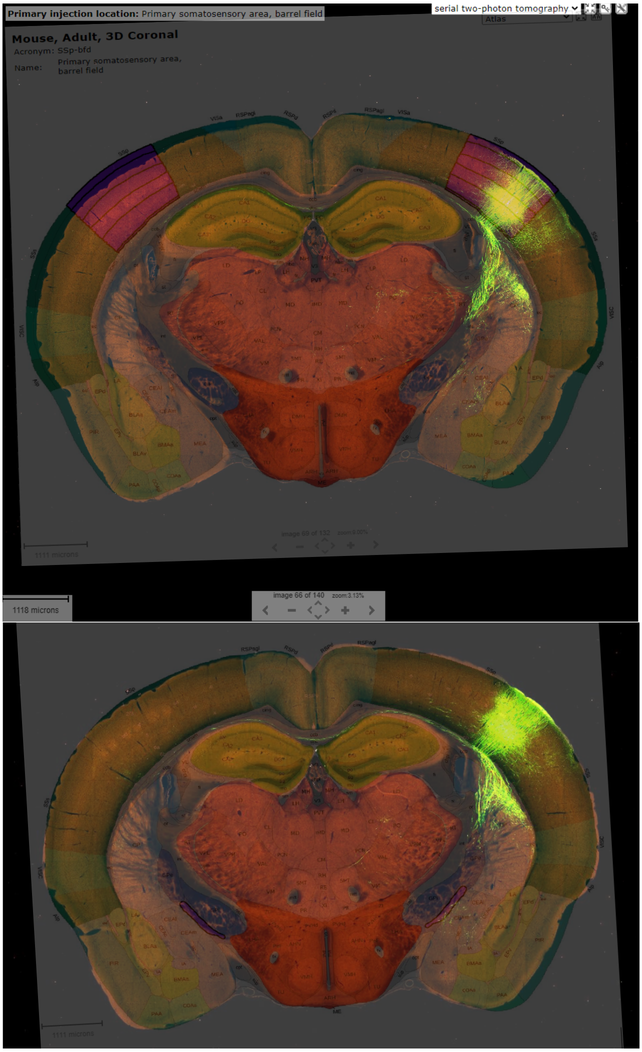

Supplemental Fig. 21 **a)** Injection structure bfd to **b)** SI. Experiment number 159602992, line Cart-Tg1-Cre. The shortcomings of experimental procedures are addressed in the discussion.

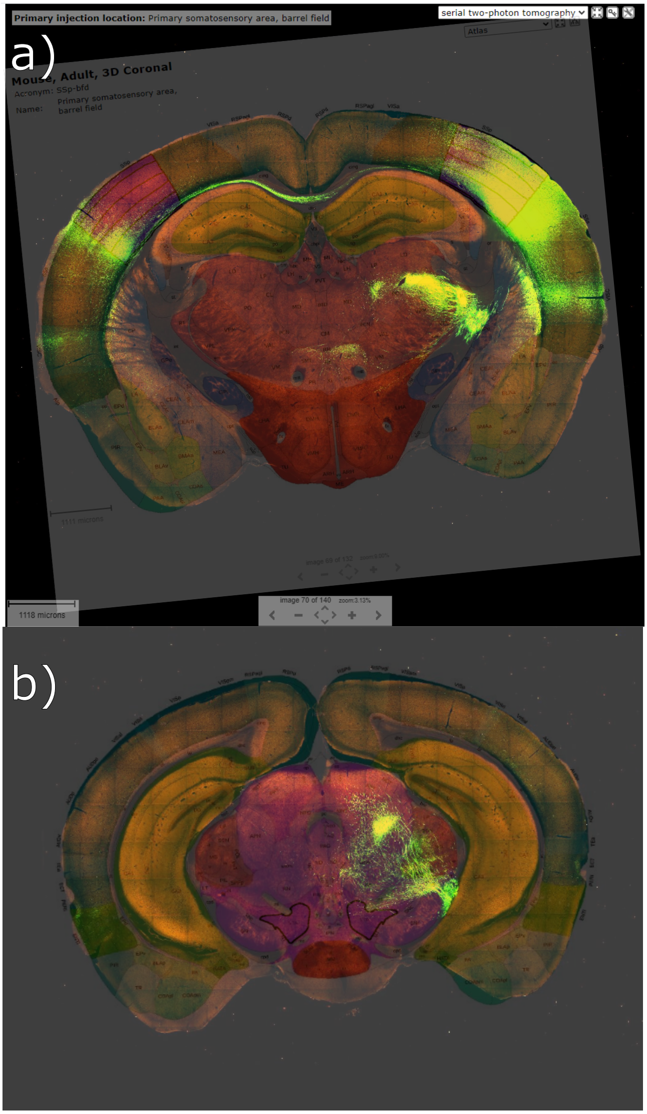

Supplemental Fig. 22 a) Injection structure bfd to **b)** VTA. Experiment number 112951804, wild-type. The shortcomings of experimental procedures are addressed in the discussion.

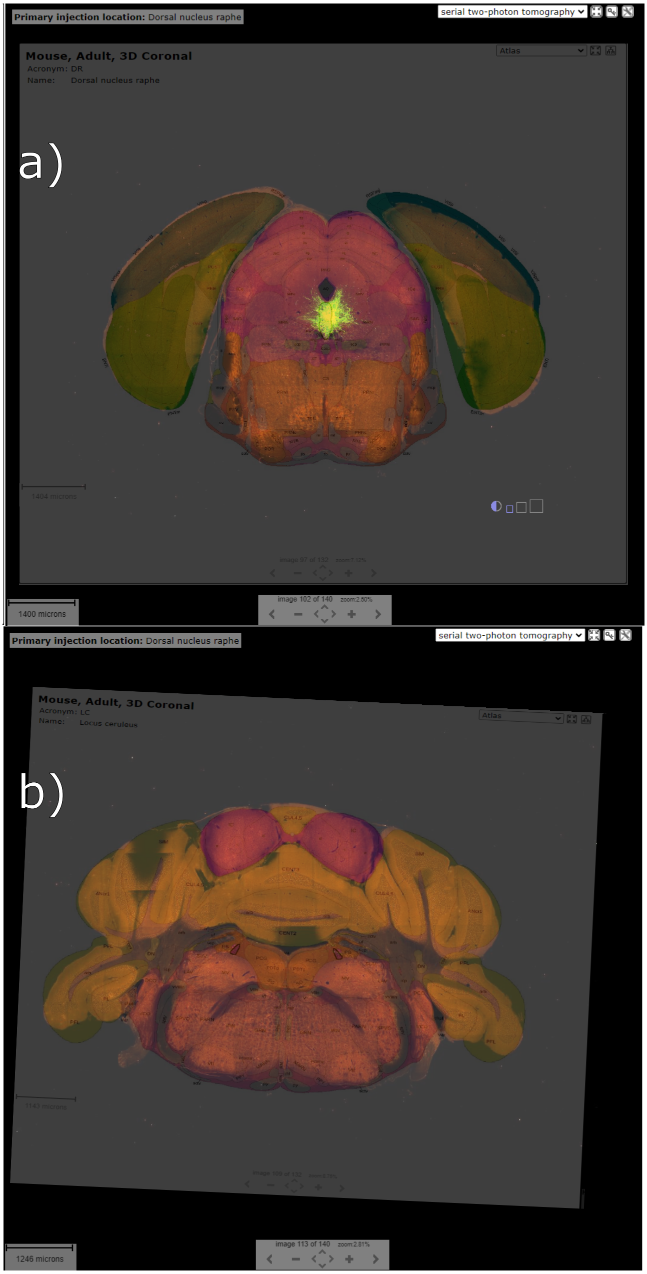

Supplemental Fig. 23 a) Injection structure DR to **b)** LC. Experiment number 547510030, Slc17a8-IRES2-Cre. The shortcomings of experimental procedures are addressed in the discussion.

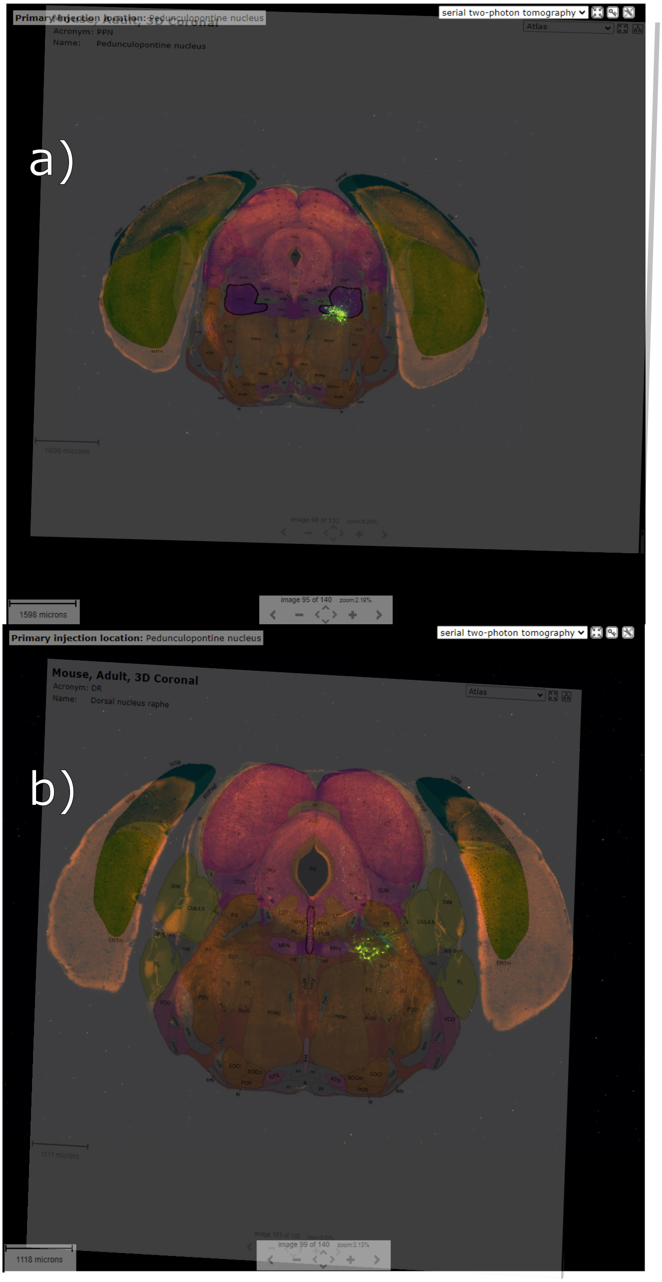

Supplemental Fig. 24 **a)** Injection structure PPN to **b)** DR. Experiment number 264566672, Chat-IRES-Cre-neo. The shortcomings of experimental procedures are addressed in the discussion.

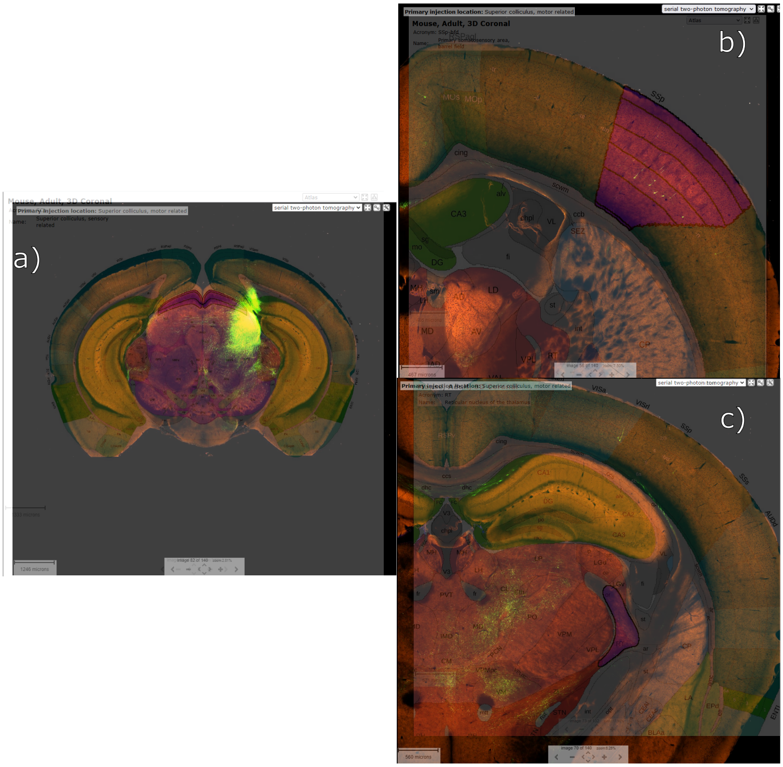

Supplemental Fig. 25 **a)** Injection structure SC to **b)** bfd and **c)** DR. Experiment number 146078721, wild-type. The shortcomings of experimental procedures are addressed in the discussion.

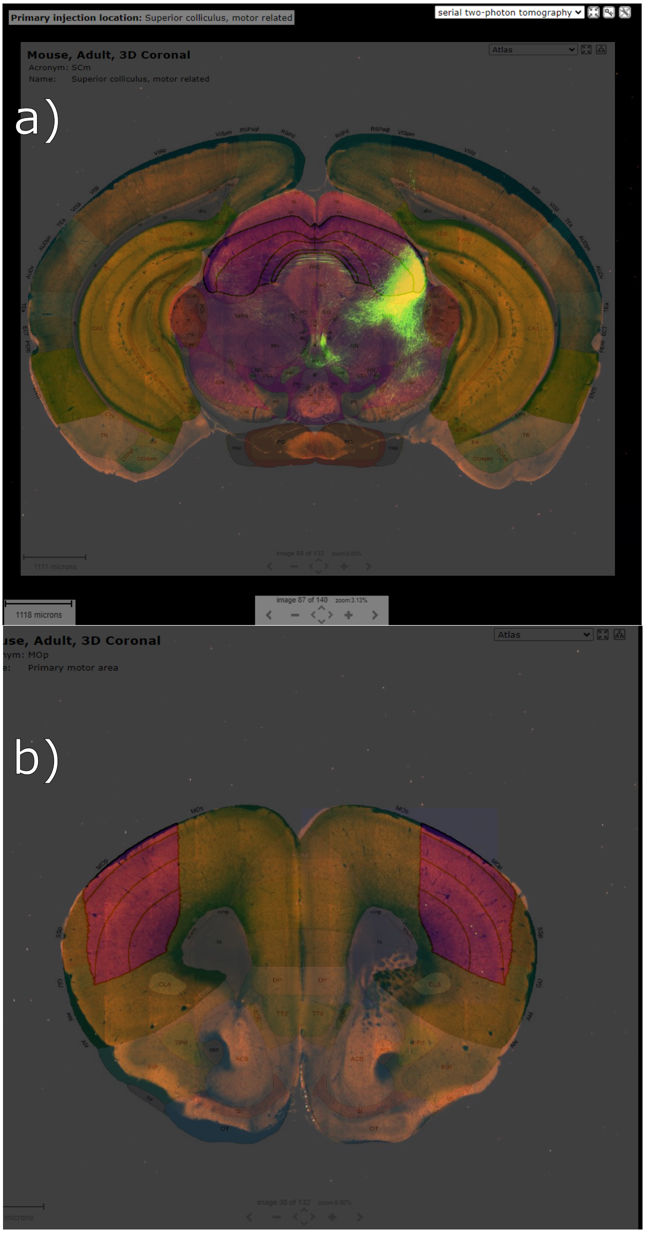

Supplemental Fig. 26 **a)** Injection structure SC to **b)** MOp. Experiment number 175158132, wild-type. The shortcomings of experimental procedures are addressed in the discussion.

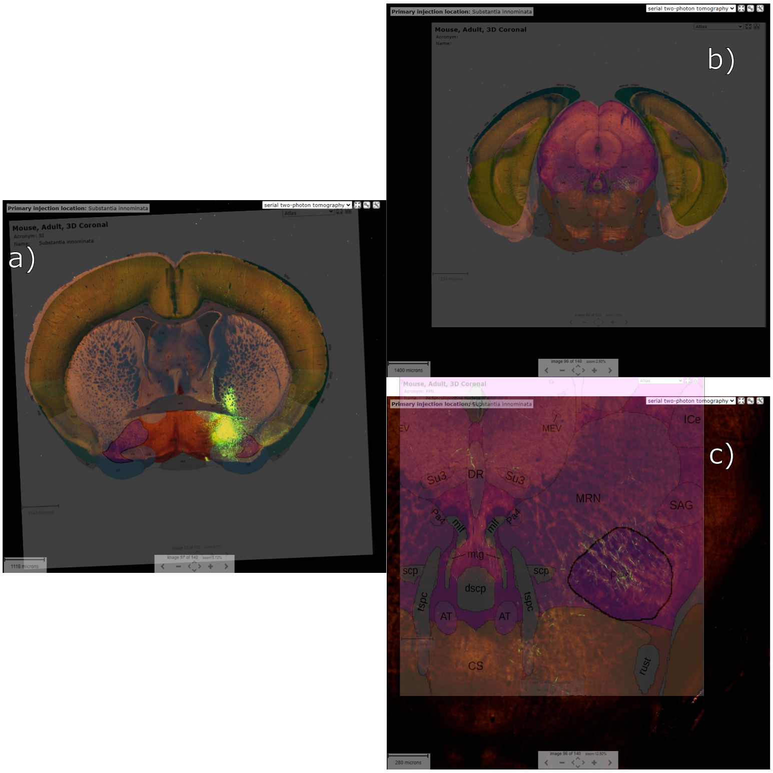

Supplemental Fig. 27 **a)** Injection structure SI to **b)** PPN. **c)** zoom on PPN. Experiment number 305026861, Drd3-Cre-KI196. The shortcomings of experimental procedures are addressed in the discussion.

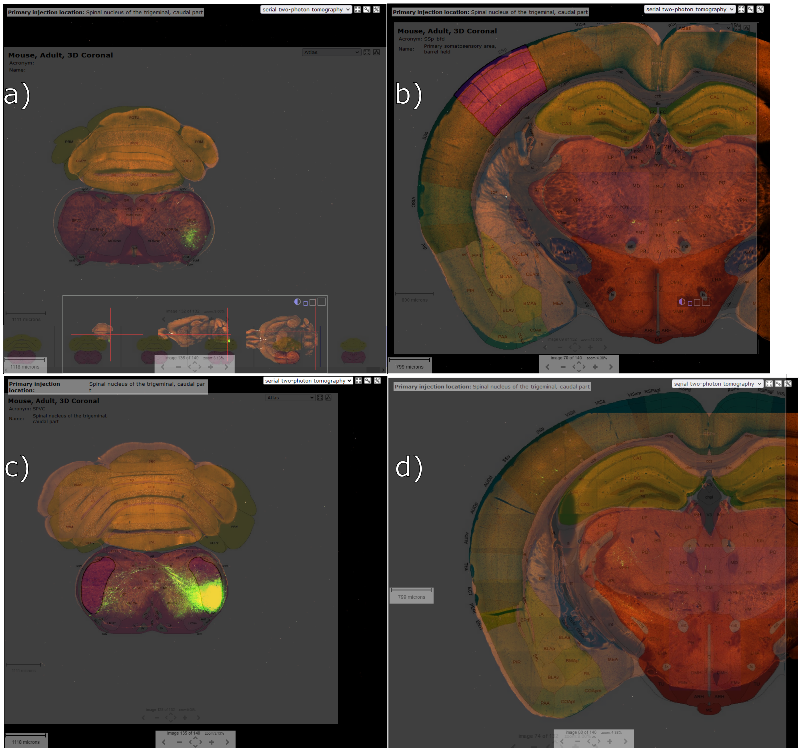

Supplemental Fig. 28 **a)** Injection structure SPVc to **b)** bfd. **c)** Injection structure SPVc to **d)** bfd. a) and b) experiment number 287460307, Cck-IRES-Cre. c) and d) experiment number 147215105, wild-type. The shortcomings of experimental procedures are addressed in the discussion.

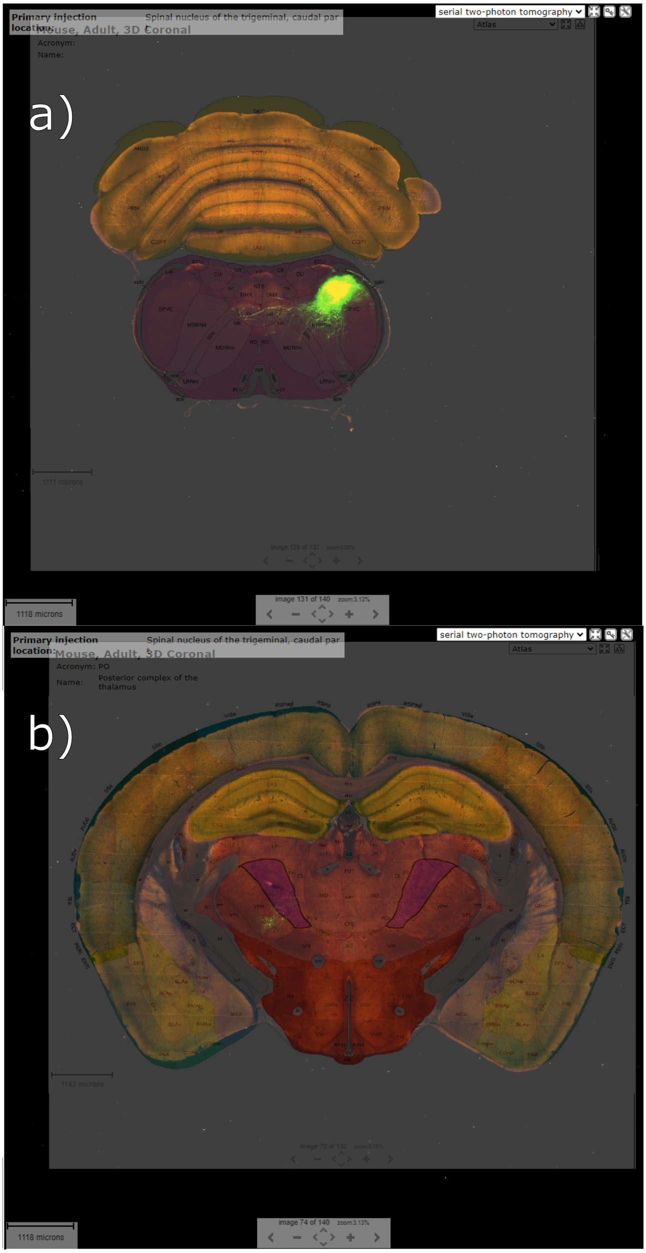

Supplemental Fig. 29 **a)** Injection structure SPVc to **b)** PO. Experiment number 114402050, wild-type. The shortcomings of experimental procedures are addressed in the discussion.

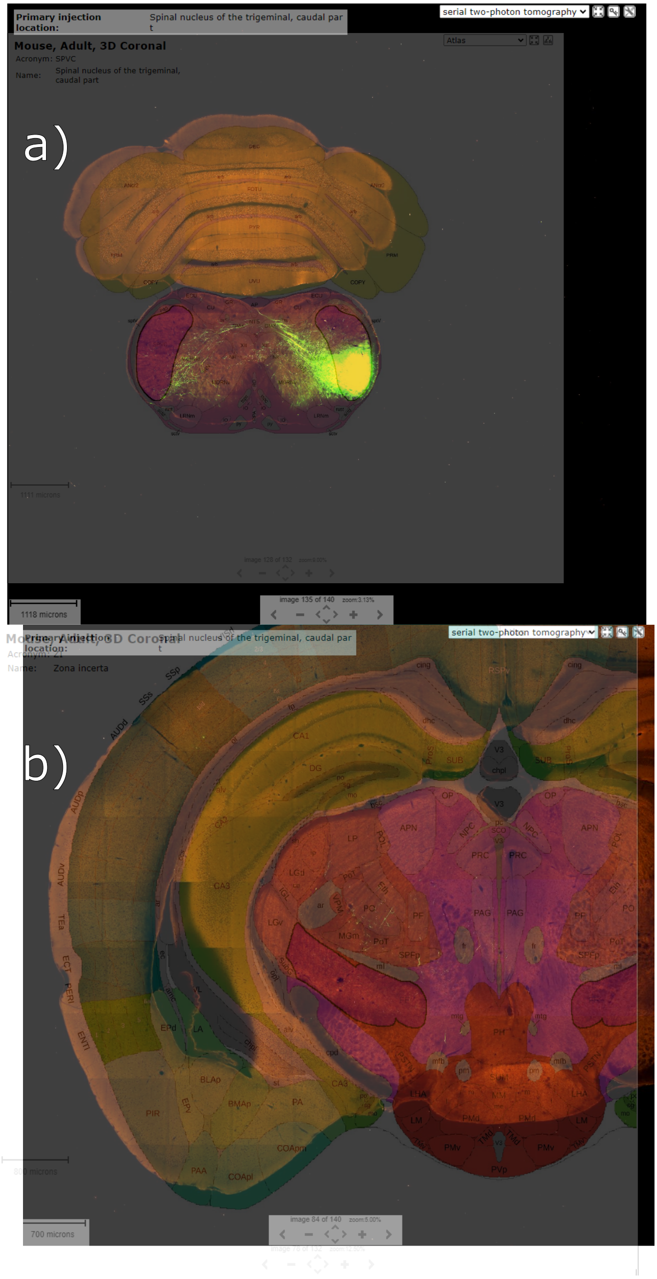

Supplemental Fig. 30 a) Injection structure SPVc to **b)** ZI. Experiment number 147215105, wild-type. The shortcomings of experimental procedures are addressed in the discussion.

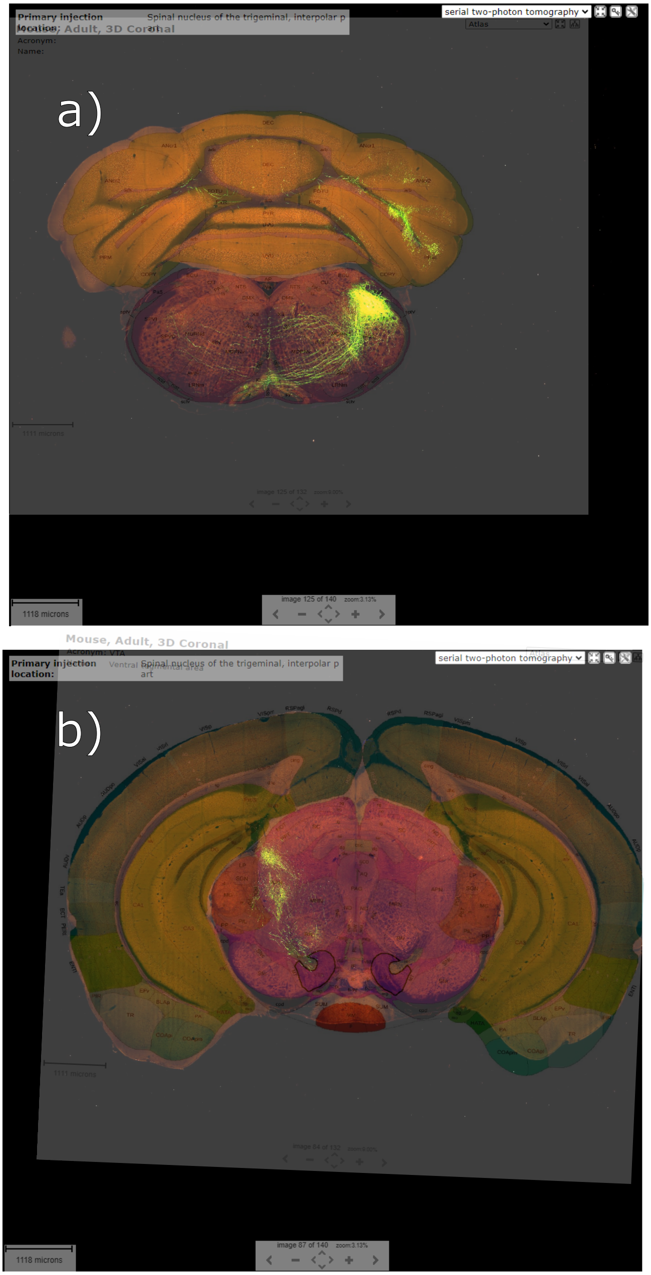

Supplemental Fig. 31 **a)** Injection structure SPVi to **b)** VTA. Experiment number 272738620, wild-type. The shortcomings of experimental procedures are addressed in the discussion.

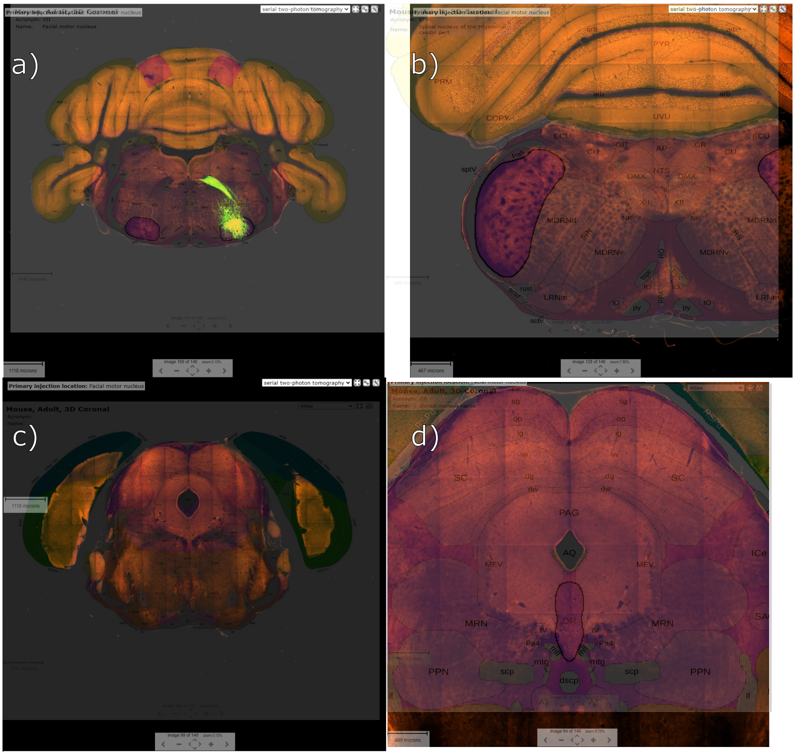

Supplemental Fig. 32 **a)** Injection structure VII to **b)** SPVc, **c)** SC and **d)** DR. Experiment number 113766744, Grik4-Cre. The shortcomings of experimental procedures are addressed in the discussion.

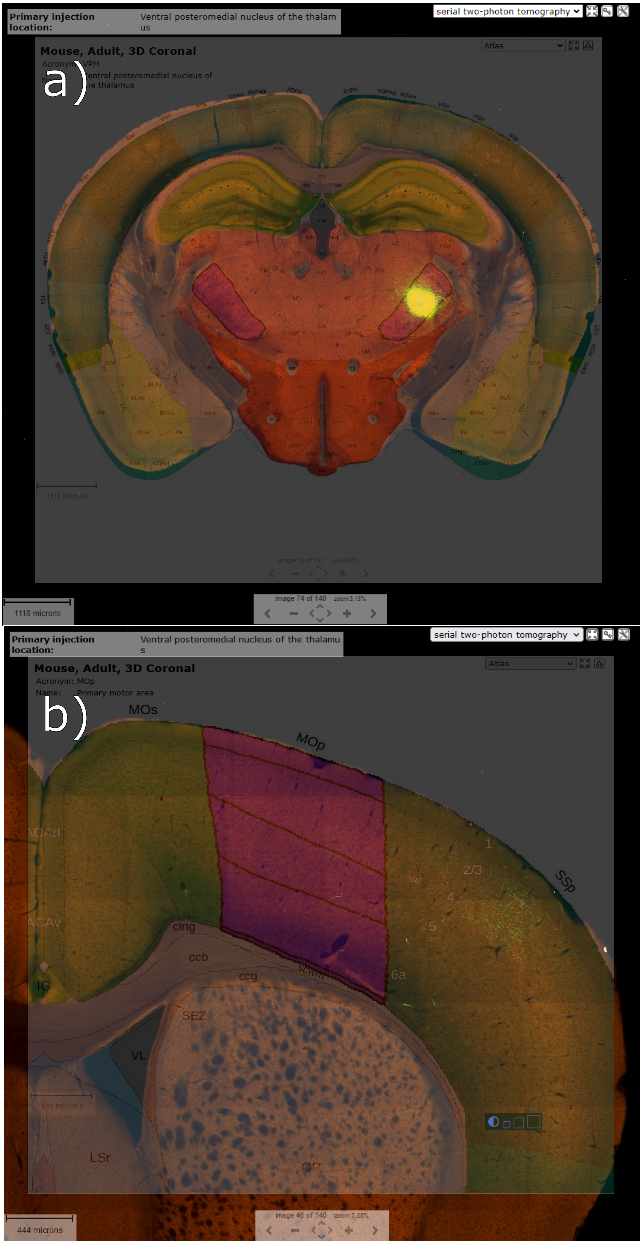

Supplemental Fig. 33 **a)** Injection structure VPM to **b)** MOp. Experiment number 312240825, Slc17a6-IRES-Cre. The shortcomings of experimental procedures are addressed in the discussion.

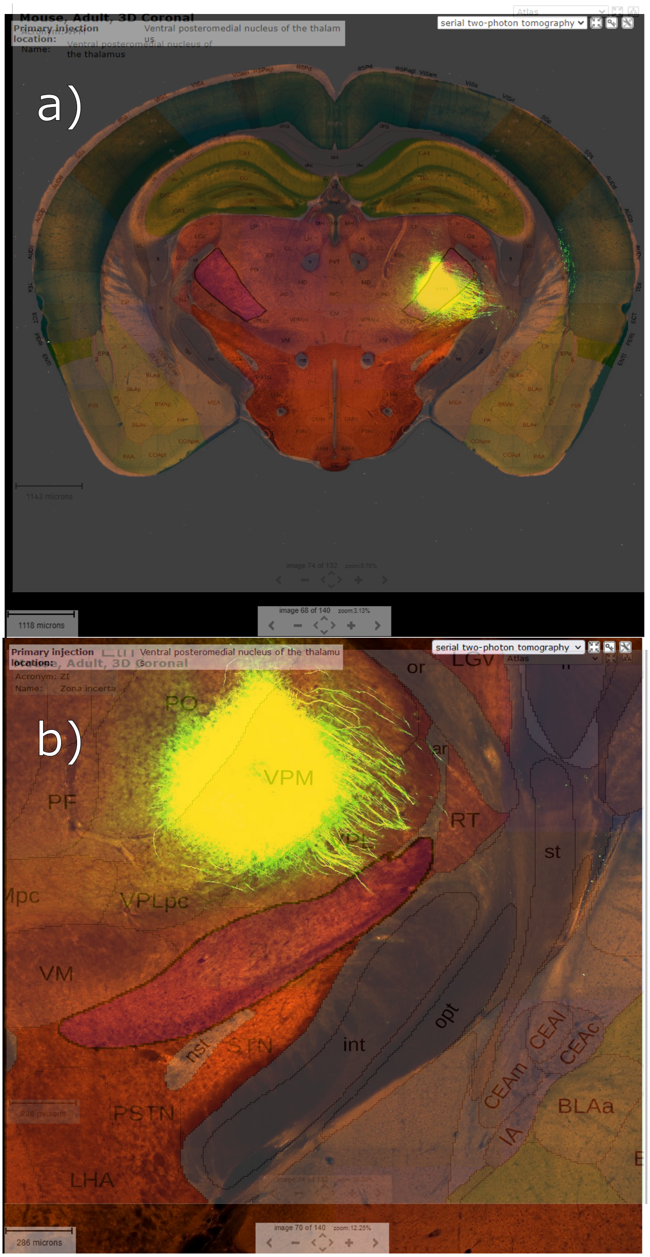

Supplemental Fig. 34 a) Injection structure VPM to **b)** ZI. Experiment number 268399868, Ppp1r17-Cre-NL146. The shortcomings of experimental procedures are addressed in the discussion.

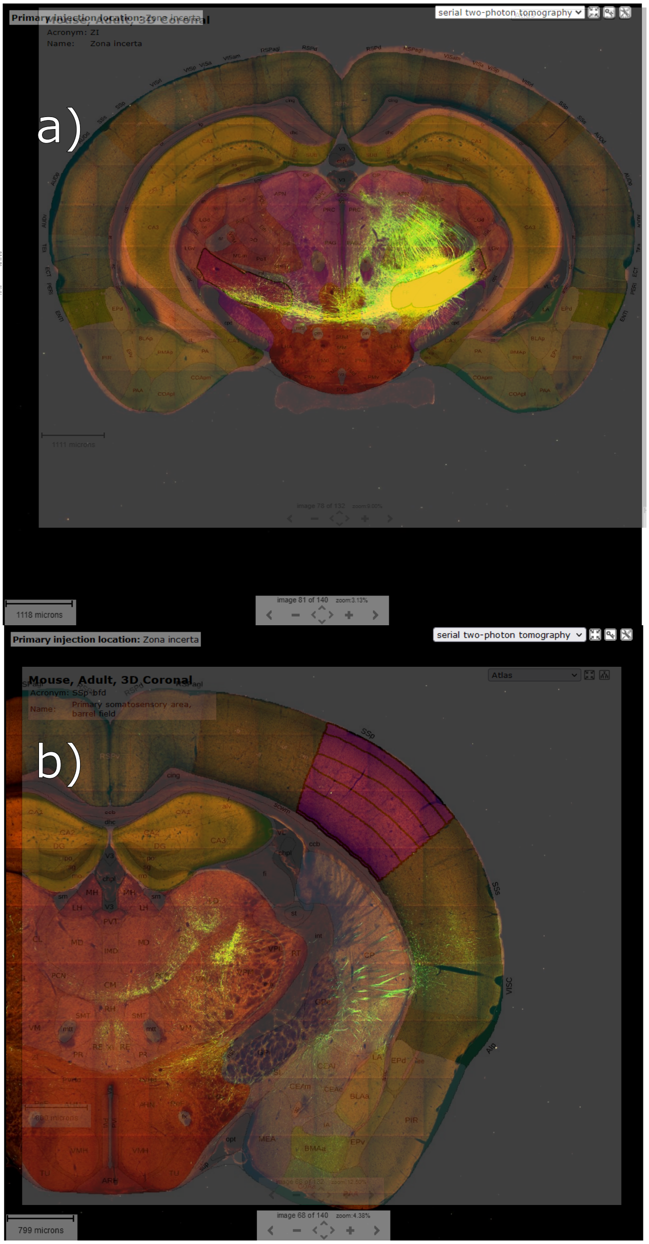

Supplemental Fig. 35 **a)** Injection structure ZI to **b)** bfd. Experiment number 113095845, wild-type. The shortcomings of experimental procedures are addressed in the discussion.
